# Effect of Micronutrients and L-Carnitine as Antioxidant on Sperm Parameters, Genome Integrity, and ICSI Outcomes: Randomized, Double-Blind, and Placebo-Controlled Clinical Trial

**DOI:** 10.3390/antiox12111937

**Published:** 2023-10-31

**Authors:** Marwa Lahimer, Oumaima Gherissi, Nesrine Ben Salem, Henda Ben Mustapha, Véronique Bach, Hafida Khorsi-Cauet, Hedi Khairi, Habib Ben Ali, Moncef BenKhalifa, Mounir Ajina

**Affiliations:** 1 Service of Reproductive Biology, University Hospital Farhat Hached, Sousse, University of Sousse, Sousse 4000, Tunisia; gherissi.oumaima@gmail.com (O.G.); nesrinebensalem@hotmail.com (N.B.S.); henda_mustapha@live.fr (H.B.M.); 2 Exercise Physiology and Physiopathology: From Integrated to Molecular “Biology, Medicine and Health” (Code: LR19ES09), Sousse 4002, Tunisia; 3 PERITOX-(UMR-I 01), UPJV/INERIS, UPJV, CURS, Chemin du Thil, 80025 Amiens, France; veronique.bach@u-picardie.fr (V.B.); hafida.khorsi@u-picardie.fr (H.K.-C.); benkhalifa.moncef@chu-amiens.fr (M.B.); 4 ART and Reproductive Biology Laboratory, University Hospital and School of Medicine, Picardie University Jules Verne, CHU Sud, 80025 Amiens, France; 5 Faculty of Medicine Ibn Eljazzar of Sousse, Department of Obstetrics & Gynecology Sousse University, Sousse 4000, Tunisia; hedi.khairi@rns.tn; 6 Laboratory Histology Embryologiy, Faculty of Medicine Sousse, University of Sousse, Sousse 4000, Tunisia; alihabibsaoudite@yahoo.fr

**Keywords:** antioxidant, oxidative stress, human sperm quality, DNA integrity, ICSI outcome

## Abstract

The evaluation of sperm DNA integrity is recommended in the sixth edition of the 2021 World Health Organization guidelines. Oxidative stress has been identified as a crucial factor leading to genome decay, lipid peroxidation, and nucleoprotein oxidation. This double-blind, placebo-controlled clinical trial aimed to assess the effect of oral antioxidant treatment (Fertilis), which contains L-carnitine and some micronutrients, in the improvement of conventional sperm parameters, sperm DNA integrity and in vitro fertilization/intracytoplasmic sperm injection (IVF/ICSI) outcomes. A total of 263 participants were enrolled and randomly divided into two groups: 131 participants received the antioxidant treatment, while 132 participants received a placebo. The male partners in both groups underwent the antioxidant treatment or the placebo for a duration of three months. For each participant, we performed a hormonal test, an infectious test, a spermogram, a TUNEL assay for sperm DNA fragmentation, a toluidine blue staining for sperm DNA decondensation, and an IVF/ICSI procedure. Sperm characteristics analysis (volume, count, motility, and vitality), sperm DNA fragmentation, and sperm DNA decondensation were assessed and compared to the results preceding the antioxidant treatment. The study outcome revealed a significant decrease in the DNA fragmentation index and a significant increase in sperm motility after 3 months of treatment (*p* = 0.01 and *p* = 0.02, respectively). Additionally, a significant improvement in clinical pregnancy rate (*p* = 0.01) and life birth rate (*p* = 0.031) was observed. No significant changes were observed in conventional sperm parameters (volume, count, and vitality) or sperm DNA decondensation (SDI). Antioxidant therapy has a beneficial impact on achieving pregnancy, whether through spontaneous conception or assisted reproductive procedures (ART).

## 1. Introduction

The sixth edition of the World Health Organization estimated that approximately 17–20% of couples experience difficulty conceiving after one year of regular unprotected intercourse [1]. Infertility affects approximately 48 million couples and around 186 million individuals worldwide [2]. In 20~30% of cases, infertility can be attributed solely to a male factor [3,4]. 

Various factors can influence male fertility, including lifestyle changes [5], endocrine disruptors [6,7,8], and paternal age [9,10]. A recent study published by Lahimer et al., 2023 has reported that pesticide exposure leads to a decline in sperm quality, especially in sperm vitality and motility (type A + B), and it also results in increased sperm DNA fragmentation in the pesticide-exposed group [11]. One month later, Lahimer et al. 2023 showed the negative impact of advanced paternal age above 40 years on sperm DNA fragmentation and epigenetic changes [12]. 

Abundant evidence widely reports that oxidative stress is recognized as one of the major contributors to male infertility [13,14,15]. The first proof was provided by the pioneering research of Thaddeus Mann et al. from Cambridge University as early as 1979 [13]. The male reproductive system is particularly vulnerable to oxidative stress, mainly due to the abundant presence of polyunsaturated fatty acids in sperm membranes and especially the relatively limited antioxidant defense mechanisms within the testicles [15,16,17]. 

Previous studies have shown that oxidative stress can lead to protein oxidation, lipid peroxidation, sperm DNA damage, and impaired sperm fertilizing ability [18]. It can cause semen quality decline, including sperm motility (asthenozoospermia), reduced sperm count (oligospermia), and abnormal sperm morphology (teratozoospermia) [17]. These factors can significantly increase the risk of male infertility. Several research studies have consistently demonstrated that infertile men frequently exhibit elevated levels of oxidative stress markers, including reactive oxygen species (ROS), lipid peroxidation products (malondialdehyde, MDA), and reduced antioxidant capacity in their semen and reproductive tissues, compared to fertile men [14,16]. Therefore, antioxidant therapies have been explored to reduce the damage of oxidative stress on male fertility [19,20,21,22,23,24,25]. The systematic review and meta-analysis study of Garolla reported that Dietary supplements for male infertility have potential efficacy in improving sperm parameters and DNA integrity even though many supplements have uncertain effects [26]. Scaruffi et al., in 2021, indicated that administering antioxidant treatment to men with low fertilization rates in their previous intracytoplasmic sperm injection (ICSI) cycles could potentially have a positive impact on the reproductive potential of their sperm [27].

A network meta-analysis of randomized controlled trials (RCTs) included a total of 23 RCTs with 1,917 patients and 10 types of antioxidants. It revealed that L-carnitine exhibited the most favorable outcomes in terms of sperm motility and morphology, while Omega-3 fatty acids ranked first in improving sperm concentration. Additionally, Coenzyme-Q10 demonstrated superior effectiveness in treating both sperm motility and concentration [23]. Cavallini et al., 2012 reported the effect of 3-month therapy with 1 g *L*-carnitine and 500 mg carnitine given twice daily and 30 mg Cinnoxicam tablet every 4 days. The results of this study showed reduced levels of sperm aneuploidy associated with increased sperm count, improved biochemical/clinical pregnancies, and live births in patients with severe idiopathic oligoasthenoteratospermia [28]. 

Clinical decision making and developing evidence-based guidelines need a comprehensive sight for the effectiveness of antioxidant components, such as L-carnitine, L-glutathione, Coenzyme Q10, selenium, and zinc, to maximize therapeutic benefits on male fertility [29]. Ongoing research has demonstrated the clinical efficacy and the effect of each antioxidant’s components [24]. Adeoye et al. (2018) reported that L-glutathione had been associated with the process of fertilization and the initial development of embryos [30]. A Chinese study involving a total of 20 boars has shown the ameliorative effect of l-arginine in improving semen quality [31]. Furthermore, L-carnitine and coenzyme Q10 improve sperm motility and overall sperm quality, particularly in cases of asthenozoospermia [32]. Moreover, another Chinese study evaluated a total of 30 weaned male mice and reported that selenium deficiency induces the activation of apoptosis and impairs the reproductive system [33]. In addition, a systematic review and meta-analysis demonstrated that zinc and folic acid improved sperm motility, sperm DNA integrity, ICSI outcome, and reduced chromosomal abnormalities [34,35].

The scientific literature strongly supports the link between male infertility and oxidative stress, which has a considerable influence on sperm function and fertility. However, further research is required to better understand the underlying mechanisms since the efficacy of antioxidant supplementation in improving fertility outcomes is still a subject of debate, as some studies have shown positive effects while others have not [27].

The main objective of this double-blind, randomized clinical trial was to investigate the effectiveness of micronutrient combination on conventional sperm parameters, sperm DNA integrity, including sperm DNA fragmentation, and chromatin compaction; then, it will be possible to prove the beneficial effect of this antioxidant treatment on ICSI/IVF outcome, embryonic quality, and pregnancy achievement.

## 2. Materials and Methods

### 2.1. Study Design and Patients

This study was a randomized, double-blind, placebo-controlled clinical trial that treated a population of 263 couples consulting for male infertility between April 2020 and December 2022. The Fertilis study has been registered on Clinicaltrials.gov under NCT04193358 since July 2021. The patients were recruited from the Reproductive Medicine Unit of Farhat Hached University Hospital (Sousse, Tunisia). All patients had to undergo hormonal and infectious analyses before the treatment. The randomized participants needed to fulfill special selection criteria (Table 1). Once eligible patients were selected, the DACIMA RANDO module of the DACIMA Clinical suite^®^ web interface was used to randomly assign patients to either the Fertilis homme^®^ or to the Placebo group. This antioxidant was produced by Medis medical laboratories. Four male participants were excluded from the study for various reasons. One couple had left Tunisia, another participant experienced an allergic reaction to the treatment, a different couple decided not to continue due to financial constraints, and the last couple divorced.

#### 2.1.1. Questionnaire Design

Eligible patients completed a comprehensive panel of questions describing their lifestyle and treatment. The questionnaire covered various aspects, including sociodemographic characteristics (age, weight, body mass index, marital status, children, employment, region, and education), medical and medication history (allergy to medication and any medications taken on a regular basis and dosage), urological history (prostatitis, epididymitis, prolonged pain or swelling of the testis, sexually transmitted infection, urethritis, urinary tract infection, blood in semen, and pain after ejaculation), surgical history (microsurgery for infertility, varicocelectomy, testis biopsy, and surgeries on penis or testis), social history (smoking habits, alcohol drinking, and profession), sexual history, antioxidant treatment, body mass index (BMI), pathology (diabetes and hypertension), and infertility type and duration.

#### 2.1.2. Ethical Approval

The study was prospectively approved by the Ethical Committee of the Faculty of Medicine, Sousse. Tunisia (Approval REC: CEFMS 191/2023). Patients eligible for the study were informed of the study’s objectives and the anticipated methodology. The informed consent of patients had to be obtained prior to their inclusion in the study, and all enrolled participants were informed and agreed about the use of a double-blind placebo formulation.

#### 2.1.3. Intervention Details

Male partners received two Fertilis (Table 2) or placebo capsules twice daily for three months.

The placebo contains lactose monohydrate, microcrystalline cellulose, and magnesium stearate. Conventional semen analysis, sperm DNA fragmentation, and chromatin decondensation were performed before and after 3 months of treatment. 

#### 2.1.4. Monitoring, Visits, and Participants Follow-Up

**Figure 1:**
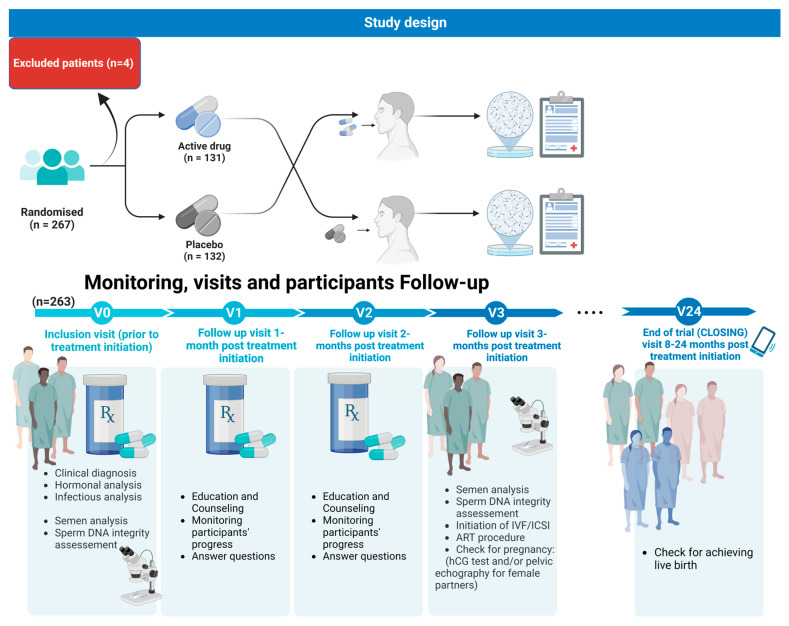
Study design. Data were collected via the DACIMA Clinical Suite^®^ web interface. The platform complies with international standards: FDA 21 CFR part 11, HIPA, ICH, MedDRA, «Health Canada», as well as the Tunisian regulations format.

At the outset of the randomization process and throughout the study’s duration, each participant’s medical history, medication usage, and supplement intake were queried. Individuals who had received antioxidant treatment during randomization were excluded from the study. All enrolled participants received clear instructions to abstain from antioxidant treatments for the entire study period. A series of follow-up visits occurred monthly over the course of three months. This monitoring procedure is described in Figure 1.

During the inclusion visit (prior to treatment initiation), V0: Patients were selected during this initial visit if they met all the inclusion criteria and none of the exclusion ones.

- Visit two was considered a follow-up visit 1 month post treatment initiation: Male patients were to be given Fertilis homme^®^ treatment or a placebo refill for a period of 1 month;
- Visit three was considered as a follow-up visit 2 months post treatment initiation. Male patients were given Fertilis homme^®^ treatment or a placebo refill for a period of 1 month;
- Visit four was considered as a follow-up visit 3 months post treatment initiation. Patients underwent semen analysis, a DNA fragmentation assay, check for pregnancy: hCG test and/or pelvic echography for the female partner; initiation of IVF/ICSI: assessment of fertilization rate, embryo cleavage rate, and embryo quality;
- Visit five: Female patients underwent a check for pregnancy (hCG test and/or pelvic echography);
- At the end of the trial (CLOSING) visit 24 months post treatment initiation, patients were asked about achieving live birth.

### 2.2. Semen Collection and Conventional Sperm Parameter Evaluation

Semen samples were obtained through masturbation after a period of abstinence lasting two to three days. Following a thirty-minute liquefaction period, a spermogram was performed to assess and quantify various sperm parameters, including volume, sperm count, motility, and vitality. The evaluation of these parameters was carried out in accordance with the criteria of the World Health Organization (WHO) in 2010.

### 2.3. Sperm DNA Fragmentation: TUNEL Assay

DNA fragmentation was performed using the TUNEL test (terminal deoxynucleotidyl transferase (TdT)-mediated dUDP nick-end labeling); it was performed using an ab66110 kit. After centrifugation and washing (10 min at 3000× *g*) to remove seminal plasma, spermatozoa were treated for 20 min with paraformaldehyde 4% to fix spermatozoa at 37 °C, and then, the semen was permeabilized in ethanol 70% for 30 min at −20 °C.

The positive control cells used were HL-60 cells treated with camptothecin (5 mL aliquot 1 × 10^6^ cells/mL), and the negative control were HL-60 cells (5 mL aliquot 1 × 10^6^ cells/mL).

The semen was washed with a wash buffer and incubated for 1 hour in a mix (reaction buffer, Br-dUTP, H_2_O_2_ sterile, TdT Enzyme). The double-helix DNA breaks were detected using mix 2 (Anti-BrdU antibody, rinse buffer) using a TUNEL kit that incubated the slides with the TdT enzyme and TUNEL solution tagging; the DUTP-tagged can occur in both the single-stranded (ss-) and double-stranded (ds-) breaks.

The slides were revealed by counting the red spermatozoa on the same day with a fluorescence microscope. On each slide, 200 spermatozoa were analyzed.

### 2.4. Chromatin Decondensation Test: Toluidine Blue Staining

DNA packaging was studied using the modified protocol of Conrad’s group for toluidine blue staining [36]. The spermatozoa were first fixed using a mixture of *v*/*v* acetone/alcohol (95%). Following fixation, they were stained using 1% TB (toluidine blue) in McIlvain buffer, which consisted of 200 mM Na_2_HPO_4_ and 100 mM citric acid at pH 3.5. The staining process took 17 minutes and was carried out at room temperature.

After staining, the slides were washed with ethanol and then mounted using the EUKiTT^®^ Quick mounting medium from Sigma. To assess the chromatin decondensation, at least 200 spermatozoa were counted under a microscope. The percentage of spermatozoa with blue-stained heads (indicating chromatin decondensation) was calculated. Up to 20% of the semen sample was considered abnormal.

### 2.5. Ovarian Stimulation and Oocyte Retrieval

The patient’s age, ovarian reserve, and previous response to stimulation played a crucial role in selecting the appropriate protocol. Younger patients with good ovarian reserve may require a standard stimulation protocol, including either the long protocol (agonist) or the short protocol (antagonist). Follicle monitoring was then important to assess the growth of the ovarian follicles using transvaginal ultrasound scans. Once at least two follicles have reached a size of 18 mm or more in diameter when the follicles are mature and contain eggs that are ready for retrieval, ovulation is triggered by administering 5000 to 10,000 IU of human chorionic gonadotropin (hCG), which mimics the luteinizing hormone surge that naturally occurs just before ovulation. The timing of hCG administration is critical, as it is scheduled when the follicles are mature and ready for egg retrieval. Therefore, transvaginal oocyte retrieval was scheduled 36 hours after the hCG injection. This procedure involves inserting a thin needle attached to an ultrasound probe through the vaginal wall in order to access the ovaries. The eggs were then aspirated from the mature follicles under ultrasound guidance.

### 2.6. ART Procedure

Following the routine protocol of the reproductive unit, as described in the study of Kacem in 2014 [37], ICSI was routinely performed on each mature oocyte at the metaphase II stage. In IVF attempts, cumulus–oocyte complexes (COCs) were inseminated with a concentration of 100,000 progressive motile sperm cells per milliliter. The injected oocytes or inseminated COCs were cultured in a suitable media overlaid with paraffin in a controlled environment at 37 °C, 6% CO_2_, and 5% CO_2_ for optimal conditions.

Fertilization assessment took place on day 1, approximately 16–18 h after insemination or injection. This assessment involved observing the presence of two pronuclei (2PN) and two polar bodies to confirm successful fertilization. On day 2 and day 3, each developing embryo was evaluated based on traditional morphological criteria, including the size and shape of blastomeres and the extent of fragmentation. Embryos were classified as “good” if they met specific criteria: (1) had equal-sized cells, (2) contained 3–5 blastomeres on day 2 and/or 6–9 cells on day 3, and (3) exhibited <20% cytoplasmic fragments on the embryonic surface. Following evaluation, one to three of the highest-scoring embryos were typically chosen for transfer to the patient’s uterus.

### 2.7. Embryo Culture 

The embryo culture step is important to provide the best possible conditions for embryo development. The embryos were cultured using sequential media (G-1™ PLUS and G-2™ PLUS; Vitrolife, Gothenburg, Sweden). On the second day following oocyte retrieval, the cleavage stage embryos underwent evaluation based on the consensus scoring system established during the Istanbul workshop of 2011 [38]. On the fifth day, a professional embryologist conducted a morphological assessment of blastocyst quality. Among the available blastocysts, 1–2 of the best ones were chosen for intrauterine transfer. The morphological grading followed the classification system established by Gardner and colleagues [39]. Blastocysts with a 3–5 expansion degree, along with trophectoderm and inner cell mass of grad B4 and B3, or a combination of B4 and B3, were categorized as top-quality blastocysts. Any other blastocysts that did not meet these criteria were classified as non-top blastocysts [40].

### 2.8. Embryo Transfer

The embryo transfer is an important step to optimize the chances of successful implantation and a healthy pregnancy. The timing of the embryo transfer depends on factors like embryo quality, patient age, and medical history. According to our standard embryo transfer protocol, it can be performed on days 2, 3, or 5 after fertilization. Prior to the embryo transfer, the uterus of the patient is prepared to create an optimal environment for embryo implantation. This may involve using hormonal medications like progesterone to support the uterine lining. The patient was positioned in lithotomy, and a speculum was inserted to provide a clear view of the cervix. Any cervical mucus and vaginal discharge were gently cleared using sterile cotton swabs. A thin catheter containing the selected embryos was gently inserted through the cervix into the uterus, where the embryos are deposited. 

After embryo transfer, the specialist prescribed support with 400 mg/day of intravaginal progesterone. The progesterone supplement is to support the early stages of pregnancy that can be detected about 10–14 days after the embryo transfer. A pregnancy test was conducted to determine if the procedure was successful. Ultrasound imaging confirmed clinical pregnancy 6–8 weeks post-embryo transfer.

### 2.9. IVF-ICSI Outcomes

Embryonic quality assessment was determined by calculating the following parameters, including maturation rate, fertilization rate, segmentation rate, and blastocyst rate, using the following formulas:

Maturation rate (%) = (number (No) of mature oocytes/No of retrieved oocytes) × 100

Fertilization rate (%) = (No of zygotes/No of mature oocytes) × 100

Cleavage rate (%) = (No of embryos/No of zygotes) × 100

Blastocyst rate (%) = (No of blastocysts/No of embryos) × 100

### 2.10. Statistical Analysis

According to the statistical plan and after comparing the clinical and lab outcomes between the two groups of the study, the results were presented as a mean ± standard deviation (SD) and percentile. The normality of continuous variables was assessed using the Kolmogorov–Smirnov test. Normally distributed quantitative variables were analyzed using a Student’s *t*-test, while non-parametric variables were analyzed using the Mann–Whitney–Wilcoxon test. Qualitative variables were analyzed with the chi-squared test. Data with a *p*-value ≤ 0.05 were considered statistically significant.

## 3. Results

The average age of male partners randomly assigned to the treatment and placebo groups was 38.94 ± 5.8 years.

In Table 3, the analyzed data are from a population-based questionnaire covering various aspects that may influence infertility rates in the population. The questionnaire includes information on sociodemographic characteristics, medical and medication history, urological history, surgical history in both male and female partners, and infertility type. 

According to the results shown in Table 3, there is no significant difference between the placebo group and the treatment group in the context of the questionnaire; it demonstrates that the two groups are homogeneous with respect to the assessed factors. The demographic, medical, urological, surgical, and infertility-related characteristics, as well as lifestyle factors, appear to be similarly distributed between the two groups.

This lack of significant difference ensures that any subsequent differences observed between the groups, especially in terms of fertility outcomes, are more likely to be attributed to the treatment itself rather than pre-existing disparities in their characteristics.

### 3.1. Conventional Sperm Parameters and Sperm DNA Integrity

The conventional sperm parameters and DNA integrity were compared before and after male antioxidant therapy and compared with placebo results.

As shown in Table 4, no significant differences were found in conventional sperm parameters (volume, count, motility, and vitality) as compared with the placebo. It is worth mentioning that sperm morphology assessment and round cells were not conducted, as they were not a part of the routine practice in the assisted reproductive technology (ART) (mean of baseline sperm typical form: 9.16% ± 13.14% and round cells: 1.21 ± 1.8). The laboratory’s decision not to assess sperm morphology can be attributed to several key factors. Firstly, the prescribers typically request spermogram results rather than spermocytogram assessments. Sperm mobility and sperm count can vary over time; however, sperm morphology remains relatively stable. In the case of teratozoospermia, it is always associated with an underlying condition (such as varicocele), and as almost everybody is undergoing intracytoplasmic sperm injection, a sperm with normal morphology and, most importantly, viability is always injected. Moreover, clinicians often rely on other sperm parameters that exhibit a stronger correlation with fertility outcomes. Finally, it is well known that laboratories may adhere to established clinical guidelines and standards of practice in the field of ART, and if these guidelines do not stipulate routine sperm morphology assessment, the laboratory may follow this guidance in its approach to patient care. 

After 3 months of antioxidant treatment, sperm DNA fragmentation significantly decreased, with the DFI mean dropping from 20% to 14.5% (*p* = 0.01). There is a clear decrease in sperm DNA decondensation, but no significant differences were found after 3 months of antioxidant treatment.

### 3.2. ICSI/IVF Outcome

Table 5 represents the concentration of the reproductive hormone of the female partner in the two groups, the Fertilis and placebo groups. No significant difference was observed in hormones related to serum FSH (follicle-stimulating hormone), serum LH (luteinizing hormone), testosterone (TSH), Estradiol (E2), prolactin (PRL), and AMH (anti-Müllerian hormone). Furthermore, these concentrations remain in the normal ranges.

In the study assessing the ART (assisted reproductive technology) procedure, the average age of the female partners included in the study was 33.2, with a standard deviation (±) of 4.6 years. This means that the age of the female partners in the study ranged from approximately 28.6 to 37.8. The average time between the end of treatment and the completion of ART was 95.6 days, with no significant difference between the two groups.

Table 6 demonstrates the effect of antioxidant treatment on the ICSI outcome and IVF outcome groups. Out of the 131 males who received Fertilis treatment, 57 underwent ICSI, and 18 underwent IVF. Among the 132 males in the placebo group, 57 underwent ICSI, and 13 underwent IVF. There was no significant difference in embryonic quality between the treated group and the placebo group (*p*-value > 0.05).

Ultrasound imaging was performed at 8 weeks’ gestation. A viable clinical pregnancy is achieved when a fetal heartbeat is detected. As shown in Table 7, The administration of antioxidant treatment for three months resulted in a significant improvement in the pregnancy rate (spontaneous and assisted pregnancy, *p* = 0.04) and live birth (*p* = 0.031) compared to the placebo group. Among the overall 20% pregnancy rate, 19.8% were clinical pregnancies (*p* = 0.01), two twins, and 22.9% were biochemical pregnancies.

In this study, a follow-up was conducted with the couples at the end of the procedure, and the live birth rate was calculated and compared with the placebo group. The variations in the follow-up period of the participants for determining live births are presented in the Supplementary Material. The results show an important live birth rate in the 3-month antioxidant-treated group compared to the placebo one.

The embryonic characteristics were assessed in two groups: one with pregnancy and the other without pregnancy. The statistical results demonstrated a significant rise in the number of mature oocytes, two PN zygotes, total embryos, maturation rate, and cleavage rate in the pregnancy group, as indicated in Table 8. The fertilization rate revealed an impressive increase of almost 40% in the pregnancy group compared to the non-pregnancy group with *p* < 0.0001.

## 4. Discussion

As far as is known, the current randomized, double-blind, placebo-controlled clinical trial study has been the first one to investigate the effect of a 3-month antioxidant treatment called “Fertilis” on the conventional sperm parameters and the sperm DNA integrity, including DNA fragmentation and chromatin compaction in relation to the embryonic quality and ICSI/IVF outcome. The antioxidant treatment was a combination of micronutrients based on l-carnitine and certain metabolites, including coenzyme 10, zinc, and selenium.

The results of the present study indicated a significant increase in sperm motility after three months of treatment compared to the baseline. These results are consistent with a prospective controlled study published in 2022, which involved 50 patients with idiopathic oligoasthenozoospermia (OA) and 35 fertile controls. The participants received a daily oral dose of 300 mg of coenzyme Q10 (CoQ10) over a three-month period. Indeed, the results demonstrated that CoQ10 treatment led to improvements in sperm progressive motility, total motility, seminal antioxidant markers, and seminal CoQ10 levels [41]. A network meta-analysis included 23 randomized controlled trials and reported that L-carnitine and coenzyme Q10 have a strong ameliorative effect on sperm motility [22]. Several studies confirmed these results [42,43,44,45,46]. 

In the present study, sperm characteristics showed no significant improvement compared to the placebo group. These results agree with the work of Tunc et al., 2009 and Tremellen et al., 2007. Indeed, these authors studied a total of 56 people treated with the antioxidant Mene-vit^®^ ADAM for three months. Their results did not reveal any significant improvement in sperm parameters, including sperm concentration, motility, and morphology. They did not note any beneficial effect on oocyte fertilization rate or embryo quality [47,48]. The study was published by Kacem in 2014 in the context of which a total of 48 infertile couples were given Fertimax2 antioxidant treatment for a minimum duration of two months. The results did not show any significant difference in sperm parameters between the treated group and the control group [37]. Conversely, the study published by Yaris in 2021 involved 122 patients with idiopathic infertility and revealed that two combinations of antioxidants had a positive impact on sperm parameters. The first combination included l-carnitine (1 g), acetyl-l-carnitine (0.5 g), fructose (1 g), citric acid (0.50 mg), selenium (50 µg), coenzyme Q10 (20 mg), vitamin C (90 mg), zinc (10 mg), folic acid (200 µg), and vitamin B12 (1.5 µg). The second combination consisted of l-carnitine (500 mg), selenium (50 µg), coenzyme Q10 (20 mg), vitamin C (60 mg), zinc (15 mg), folic acid (400 µg), vitamin E, and ginseng (15 µg). Both combinations were administered for a period of 6 months [49]. It is well known that certain antioxidants and nutrients have been studied for their potential effects on the fertilizing power of sperm [19,24]. Systemic reviews and meta-analyses analyzed 29 studies and demonstrated that antioxidant supplementation based on carnitines, vitamins E and C, N-acetyl cysteine, coenzyme Q10, selenium, zinc, folic acid, and lycopene has an ameliorative effect on semen parameters, assisted reproductive technology outcome, and birth rate [24]. Omega-3 fatty acids were ranked first for improving sperm concentration [22]. A meta-analysis study involving 149 men in the coenzyme Q10 group and 147 men in the placebo group reported that coenzyme Q10 has an ameliorative effect on sperm concentration and sperm motility [50]. In addition, an experimental study included 60 ejaculates from different boars and evaluated the effects of myo-inositol treatment on frozen–thawed sperm. The results indicated that myo-inositol at 0.5 mg/mL improved sperm motility, acrosome integrity, and fertilization ability. This treatment reduced the expression of pro-apoptotic genes. It has a beneficial effect on the concentrations, the viability, the reduction of apoptosis, and the reduction of the levels of ROS. Overall, myo-inositol during cryopreservation enhanced sperm quality fertility rates and reduced apoptosis and ROS levels in boar semen [25]. 

In the current study, the sperm morphology was not assessed, as it was not part of the routine practice in assisted reproductive technology. Abnormal sperm morphology can impact their ability to successfully fertilize an egg, influencing both natural conception and the results of procedures such as IVF and ICSI. A study conducted by Cassuto et al., 2022 involved a total of 16 couples enrolled for IVF protocol and revealed a significant correlation between morphology and gene expression in spermatozoa, a new tool to explore male infertility [51]. The antioxidant treatment can have an ameliorative effect on the sperm morphology. Mounting evidence indicated that antioxidant treatments can improve sperm morphology by reducing oxidative stress and its detrimental effects on sperm [51,52].

As far as is known, a successful pregnancy depends on both good embryonic quality and good sperm DNA integrity. These two factors play an essential role in the conception process and the proper development of the embryo [53]. The diagnosis of male infertility often relies on conventional analysis of sperm parameters, such as sperm count, motility, and morphology [54]. Agarwal, in 2005, reported that 15% of infertile patients had a normal spermogram [55]. Although these parameters provide valuable information about sperm health and function, they may not fully explain the complexity of male fertility potential [56]. The sixth edition of the World Health Organization recommended advanced diagnostic tests, such as sperm DNA fragmentation analysis, to supplement routine analysis [1]. 

To fully understand the factors affecting male infertility, advanced diagnostic tests, including sperm DNA fragmentation and sperm DNA decondensation, were assessed before and after 3 months post-antioxidant treatment. Our statistical data did not show any significant difference between the Fertilis-treated group and the placebo-treated group; on the other hand, the DFI, before and after 3 months of treatment, showed a significant decrease in the DNA fragmentation of the sperm. The DFI went from 20% (grey zone DFI = 20 to 30%: DFI is not ideal) down to less than 15% (green zone, DFI < 20%: no apparent problem) [57]. The antioxidant treatment has an ameliorative effect on single or double DNA breaks. The same result was reported by Delbarba in 2020 in a total of 60 idiopathic infertile men with high DFI. These men received a nutraceutical formulation comprising myoinositol, alpha-lipoic acid, coenzyme Q10, selenium, zinc, and B vitamins for three months. The result showed a significant increase in sperm vitality and a greater decrease in sperm DNA fragmentation [58]. A randomized, double-blind, placebo-controlled trial treated 77 men from infertile couples for three months and six months with placebo/antioxidant treatment. The study outcome reveals that three-month and six-month antioxidant treatments have no effect on sperm DFI [20]. Conversely, another double-blind, randomized, placebo-controlled trial using Proxeed plus^®^ for six months reported an ameliorating effect on progressive motility, vitality, and sperm DNA fragmentation [44]. In the current study, there was no significant effect on fertilization rate and chromatin compaction, unlike the study by Tunc, which reported a significant improvement in protamine compaction after antioxidant treatment [47]. 

Understanding sperm DNA damage is key to optimizing embryonic quality and reducing the risk of miscarriage in childbearing couples [59]. A systematic review and meta-analysis of studies including 2969 couples reported that sperm DNA damage is associated with a high risk of miscarriage levels, genetic disease transmission, and compromising embryonic and subsequent postnatal development [60]. The present study demonstrated a remarkable increase in pregnancy rate, including clinical pregnancy rate and live birth rate. The improvement in the DNA fragmentation index (DFI) may be associated with the higher pregnancy rate observed in the Fertilis group compared to the placebo group. The clinical decision of intracytoplasmic sperm injection (ICSI) showed a significant rise in the placebo group compared to the Fertilis group, which can be attributed to enhanced sperm motility. A similar result was demonstrated by Kacem et al. 2014, in a couple treated with the antioxidant Fertimax [37]. 

The statistical analysis revealed no significant improvement in embryonic quality between the Fertilis group and the placebo group. However, comparing the embryonic quality according to obtaining a pregnancy, a very significant increase in the embryonic quality was observed in the group having obtained a pregnancy. Embryo quality can vary significantly even when comparing embryos within the same treatment group. This inherent variability can create difficulties in identifying substantial improvements [61]. 

Research in this area is ongoing, and understanding the precise mechanisms by which antioxidant treatment improves pregnancy outcomes may require more investigation. In the present study, several postulates based on growing evidence can be considered. 

Postulate 1: The antioxidant treatment can affect cellular signaling pathways that are involved in embryo development and implantation. These mechanisms might not directly influence semen quality measures [62].

Postulate 2: The antioxidant treatment might influence hormonal balance in ways that are not directly related to semen quality but can impact reproductive success [63].

Postulate 3: In the current study, we did not quantify the ROS levels, the lipid peroxidation product (MDA), and the apoptosis product (i.e., free circulating DNA). The Fertilis treatment could have broader effects in reducing systemic oxidative stress, benefiting overall reproductive health.

Postulate 4: Antioxidant treatment could potentially induce epigenetic modifications in sperm or embryos, affecting their development and viability [64]. 

## 5. Conclusions

The study highlights the potential benefits of Fertilis antioxidant therapy on sperm motility, improved sperm DNA fragmentation, and pregnancy success. This study adds to the increasing body of evidence that supports the role of antioxidants in enhancing male fertility and highlights the importance of sperm DNA assessment. The significant improvement in pregnancy outcomes after three months of antioxidant treatment was likely attributed to a reduction in sperm DNA damage, particularly in terms of sperm DNA fragmentation. Genome integrity is a critical element that impacts the overall quality and viability of embryos. Our study recommendation is to undergo six months of treatment to improve conventional semen parameters and chromatin quality.

### Limits and Perspectives

Antioxidant therapies have garnered significant interest within the field of reproductive health. However, certain studies have failed to demonstrate amelioration in one or more parameters. To fully understand the advantages of Fertilis treatment, additional research is required to assess its impact on the portion of viable oxidized sperm, using a novel fluorescent probe known as “CellROX^®^R Orange”, and the effects on both in vivo and in vitro embryo quality. 

## Figures and Tables

**Table 1:** Selection criteria.

Inclusion Criteria	Exclusion Criteria
The trial included patients who meet all of the following criteria: - Male ≥ 20 years of age; - Attending the Department of Obstetrics and Gynecology of Farhat Hached University Hospital, Sousse, Tunisia, for consultation or semen analysis as part of infertility investigations; - Diagnosis of oligozoospermia; - Diagnosis of Asthenozoospermia (WHO 2010 definition); - Diagnosis of teratozoospermia1; - Diagnosis of idiopathic infertility; - Couple was a candidate for IUI, IVF, and/or ICSI.	Patients with at least one of the following criteria were not included in the trial: - Lactose intolerance as the placebo contains lactose; - Varicocele, whatever the grade; - Chronic disease with long-term treatment (asthma and cardiovascular disorders); - Erectile dysfunction (ED); - Aspermia (WHO 2010 definition); - Leukospermia (WHO 2010 definition); - Cryptozoospermia (WHO 2010 definition); - Azoospermia obstructive and non-obstructive; - Recent or current urogenital infections for both partners; - Endocrine (hormonal) disorders; - Central hypogonadism; - Patient currently on antioxidant supplementation; - Female partner older than 40 years of age; - Female partner with premature ovarian failure (POF), which is related to the cessation of ovarian function before the age of 40 (defined by a triad of signs: (i) amenorrhea for at least 4 months, (ii) decreased estradiol serum concentrations, and (iii) elevated follicle-stimulating hormone (FSH) serum concentrations (more than 40 IU/l in at least two samples a few weeks apart)); - Female partners who are poor ovarian responders (POR) (defined as the presence of at least two of the following three characteristics: (i) advanced maternal age (≥40 years) or any of the risk factors for POR such as pelvic infection, endometrioma, ovarian surgery, and extensive periovarian adhesions; (ii) a previous poor ovarian response (≤3 oocytes with a conventional stimulation protocol), and (iii) an abnormal ovarian reserve test (antral follicular count (AFC) of 5–7 follicles or anti-Müllerian hormone (AMH) of 0.5–1.1 ng/mL); - Female partner with diminished ovarian reserve (DOR), defined as (i) a woman with any of the risk factors for POR and/or (ii) an abnormal ovarian reserve test (AFC < 5–7 follicles or AMH < 0.5–1.1 ng/mL); - Female partner with prolactinoma; - Female partner with thyroid dysfunction; - Female partner who did not wish their partners to participate in the study.

**Table 2:** Content of the Fertilis antioxidant.

L-Carnitine	220 mg
L-arginine	125 mg
Vitamin E	60 mg
L-glutathione	40 mg
Zinc	20 mg
Coenzyme Q10	7.5 mg
Folic acid (vitB-9)	0.4 mg
Selenium	0.03 mg

**Table 3:** A comprehensive population-based study.

			Fertilis (n = 131)	Placebo (n = 132)	*p*-Value
Sociodemographic characteristics (Male partner), % (n)	BMI male partner kg/m^2^		26.14 ± 4.2	26.27 ± 3.9	
	Diabetes (%)	yes	2.3 (3)	3.8 (5)	0.4
		no	97.7 (128)	96.2 (127)	
	HTA (%)	yes	3.1 (4)	1.5 (2)	0.4
		no	96.9 (127)	98.5 (130)	
	Smoking (%)	yes	44.9 (59)	57.3 (76)	0.04
		no	55.1 (72)	42.7 (56)	
	Alcohol (%)	yes	11.6 (15)	19.1 (25)	0.09
		no	88.4 (116)	80.9 (107)	
Sociodemographic characteristics (Female partner), % (n)	BMI female partner kg/m^2^		26.18 ± 4.5	26.15 ± 4.2	
	Diabetes (%)	yes	2.3 (3)	1.5 (2)	0.1
		no	97.7 (128)	98.5 (130)	
	HTA (%)	yes	0	0	-
		no	100	100	
	Smoking (%)	yes	0.8 (1)	0.8 (1)	0.9
		no	99.2 (130)	99.2 (131)	
	Alcohol (%)	yes	0	0	-
		no	100	100	
Male infertility, % (n)	Male urogenital pathology (%)		0	0	
	Acquired testicular pathology (%)		0.8 (1)	0.8 (1)	0.7
	Auto-antibody (%)		6.1 (8)	9.8 (13)	0.1
Female infertility, % (n)	Right fallopian tube (%): Normal Obstructed/absent/ligated Not defined		94.7 (124)	92.4 (122)	0.7
			3.8 (5)	6.1 (8)	0.7
			1.5 (2)	1.5 (2)	0.5
	Left fallopian tube (%): Normal Obstructed/absent/ligated Not defined		91.6 (120)	96.2 (127)	0.3
			6.1 (8)	2.3 (3)	0.2
			1.5 (2)	1.5 (2)	0.2
	Anovulation/dysovulation (%)		0	0	
	Ovarian reserve anomaly (%)		0.8 (1)	0.8 (1)	0.7
	Endometriosis (%)		0.8 (1)	0	0.4
	Cone biopsy (%)		0	0	
	Uterine pathology (%)		0	0.8 (1)	0.5
	Cervical pathology (%)		0.8 (1)	0	0.4
	Stenosis (%)		0	0	
	Other pathology (%)		0.8	0	0.4
Infertility type, % (n)		Primary	71.7 (94)	82.2 (109)	0.04
		secondary	28.3 (37)	17.8 (23)	

BMI: body mass index, HTA: hypertension. Values are expressed as percentile and mean ± SD.

**Table 4:** Comparative effect of Fertilis treatment and placebo in sperm parameters and sperm DNA integrity.

	Fertilis (n = 131)	Placebo (n = 132)	*p*-Value
Volume (mL)	T0 = 2.4 ± 1.5 TF = 2.8 ± 1.7 *p*-value = 0.6	T0 = 2.5 ± 1.5 TF = 2.8 ± 1.7 *p*-value = 0.5	0.7 0.5
Sperm count (10^6^ spz/mL) *	T0 = 44.6 ± 42.2 TF = 45.9 ± 38.2 *p*-value = 0.9	T0 = 53.2 ± 44.48 TF = 52.25 ± 45.58 *p*-value = 0.3	0.4 0.9
Sperm motility (%)	T0 = 20.74 ± 13.7 TF = 22.5 ± 15.35 *p*-value = 0.02	T0 = 22.9 ± 12.5 TF = 24.6 ± 12.91 *p*-value = 0.1	0.9 0.3
Sperm vitality (%)	T0 = 65.45 ± 16.83 TF = 69.6 ± 15.65 *p*-value = 0.7	T0 = 65.93 ± 16.37 TF = 70.25 ± 13.98 *p*-value = 0.9	0.8 0.7
DFI (%)	T0 = 20 TF = 14.5 *p*-value = 0.01	T0 = 18 TF = 13 *p*-value < 0.05	0.9 0.2
SDI (%)	T0 = 50 TF = 44 *p*-value = 0.4	T0 = 43 TF = 52.5 *p*-value = 0.3	0.2 0.6

Values are expressed as percentile and mean ± SD. DFI: DNA fragmentation index and SDI: decondensation index, * spz: spermatozoa.

**Table 5:** Female reproductive hormone concentrations in Fertilis and placebo groups.

	Fertilis	Placebo	*p*-Value
BMI female partner kg/m^2^	26.18 ± 4.5	26.15 ± 4.2	
FSH (UI/I)	7.5 ± 3.5	7.74 ± 3.9	0.6
LH (UI/I)	4.5 ± 3.09	5.01 ± 2.3	0.3
E2 (pg/mL)	48.11 ± 36.9	49.05 ± 24.83	0.8
PRL (ng/mL)	18.43 ± 25.02	20.82 ± 29.27	0.5
AMH (ng/mL)	2.35 ± 3.1	2.53 ± 2.4	0.6
TSH (mU/I)	2.36 ± 1.6	2.11 ± 1.1	0.2

All values are expressed as mean ± SD.

**Table 6:** Embryology data and ICSI/IVF outcomes in the treated group (Fertilis) and the control group (placebo).

ICSI/IVF Outcome	ICSI (n = 114)			FIV (n = 31)		
	Fertilis (n = 57)	Placebo (n = 57)	*p*-Value	Fertilis (n = 18)	Placebo (n = 13)	*p*-Value
No. of retrieved *COC	3.7 ± 2.9	3.9 ± 3.5	0.9	3.5 ± 2.8	2.4 ± 2.2	0.2
No. of mature Oocytes	2.7 ± 2.2	3 ± 2.9	0.9	2.4 ± 1.9	2 ± 2.3	0.3
Inseminated oocytes (*IVF)	0	0	-	2.6 ± 1.9	2.5 ± 2.4	0.7
Microinjected oocytes (*ICSI)	2.7 ± 2.3	2.9 ± 2.8	0.9	0	0	-
Zygotes 2 PN	1.7 ± 1.8	1.8 ± 2.2	0.9	1.8 ± 1.9	1.7 ± 2.1	0.7
Total embryos	1.8 ± 1.8	1.8 ± 1.9	0.9	1.6 ± 1.7	1.7 ± 1.8	0.9
Transferred embryos	2 ± 0.9	2 ± 1	0.8	2 ± 0.6	2 ± 1	0.9
Maturation rate (%)	67.8 ± 38.9	57.57 ± 35.8	0.2	68.8 ± 48.5	71.2 ± 39.1	0.5
Fertilisation rate (%)	48.13 ± 45.5	38.9 ± 33.7	0.4	48.5 ± 42.4	48.2 ± 41.1	0.9
Cleavage rate (%)	54.1 ± 49.9	44.4 ± 49	0.3	57.1 ± 46.5	57.9 ± 46.2	0.9
Blastocyst rate (%)	15.1 ± 63.3	10.5 ± 31.5	0.6	13.1 ± 34	11.9 ± 32.7	0.7

Values are expressed as percentile and mean ± SD, *IVF: in vitro fertilization, *ICSI: intracytoplasmic sperm injection, and *COC: cumulus–oocyte complex.

**Table 7:** Ameliorative effect of Fertilis on pregnancy rate achievement.

	Fertilis (n = 131)	Placebo (n = 132)	*p*-Value
Biochemical pregnancy ^α^ (%)	22.9 (30/131)	16.7 (22/132)	0.1
Clinical pregnancy ^α^ (%)	19.8 (26/131)	11.4 (15/132)	0.04
Pregnancy rate ^α^ (%)	20.6 (27/131)	9.8 (13/132)	0.01
Live birth ^α^ (%)	13 (17/131)	5.3 (7/132)	0.031
No. Twins	2	0	

Values are expressed as percentile (number) and ^α^: spontaneous + assisted pregnancy.

**Table 8:** Comparison of embryonic quality in the two groups based on the pregnancy.

	Pregnancy		*p*-Value
	YES (n = 40)	NO (n = 223)	
No. of retrieved COC	4.8 ± 3.04	3.4 ± 3.07	0.08
No. of mature Oocytes	4.8 ± 2.6	2.7 ± 2.5	0.04
Zygotes 2 PN	3.13 ± 1.7	1.47 ± 2.07	0.004
Total embryos	2.5 ± 1.06	1.29 ± 1.8	0.01
Blastocyst	1.13 ± 1.8	0.73 ± 1.9	0.4
Maturation rate	88.44 ± 15.46	65.8 ± 40.02	0.03
Fertilisation rate	86.04 ± 21.91	42.41 ± 42.76	<0.0001
Cleavage rate	89.44 ± 23.24	50.24 ± 48.11	0.002

## Data Availability

Not applicable.

## Supplementary Materials

The following supporting information can be downloaded at: https://www.mdpi.com/article/10.3390/antiox12111937/s1.

## References

[1] Boitrelle F., Shah R., Saleh R., Henkel R., Kandil H., Chung E., Vogiatzi P., Zini A., Arafa M., Agarwal A. (2021). The Sixth Edition of the WHO Manual for Human Semen Analysis: A Critical Review and SWOT Analysis. Life.

[2] Agarwal A., Parekh N., Panner Selvam M.K., Henkel R., Shah R., Homa S.T., Ramasamy R., Ko E., Tremellen K., Esteves S. (2019). Male Oxidative Stress Infertility (MOSI): Proposed Terminology and Clinical Practice Guidelines for Management of Idiopathic Male Infertility. World J. Mens. Health.

[3] Leslie S.W., Soon-Sutton T.L., Khan M.A. (2023). Male Infertility. StatPearls.

[4] Agarwal A., Mulgund A., Hamada A., Chyatte M.R. (2015). A unique view on male infertility around the globe. Reprod. Biol. Endocrinol..

[5] Montjean D., Godin Pagé M.-H., Bélanger M.-C., Benkhalifa M., Miron P. (2023). An Overview of E-Cigarette Impact on Reproductive Health. Life.

[6] Montjean D., Neyroud A.-S., Yefimova M.G., Benkhalifa M., Cabry R., Ravel C. (2022). Impact of Endocrine Disruptors upon Non-Genetic Inheritance. Int. J. Mol. Sci..

[7] Giulioni C., Maurizi V., Castellani D., Scarcella S., Skrami E., Balercia G., Galosi A.B. (2022). The environmental and occupational influence of pesticides on male fertility: A systematic review of human studies. Andrology.

[8] Daoud S., Sellami A., Bouassida M., Kebaili S., Ammar Keskes L., Rebai T., Chakroun Feki N. (2017). Routine assessment of occupational exposure and its relation to semen quality in infertile men: A cross-sectional study. Turk. J. Med. Sci..

[9] Aitken R.J. (2023). Male reproductive ageing: A radical road to ruin. Human. Reprod..

[10] Ashapkin V., Suvorov A., Pilsner J.R., Krawetz S.A., Sergeyev O. (2023). Age-associated epigenetic changes in mammalian sperm: Implications for offspring health and development. Hum. Reprod. Update.

[11] Lahimer M., Capelle S., Lefranc E., Cabry R., Montjean D., Bach V., Ajina M., Ali H.B., Benkhalifa M., Khorsi-Cauet H. (2023). Effect of pesticide exposure on human sperm characteristics, genome integrity, and methylation profile analysis. Environ. Sci. Pollut. Res..

[12] Lahimer M., Montjean D., Cabry R., Capelle S., Lefranc E., Bach V., Ajina M., Ben Ali H., Khorsi-Cauet H., Benkhalifa M. (2023). Paternal Age Matters: Association with Sperm Criteria’s- Spermatozoa DNA Integrity and Methylation Profile. J. Clin. Med..

[13] Jones R., Mann T., Sherins R. (1979). Peroxidative breakdown of phospholipids in human spermatozoa, spermicidal properties of fatty acid peroxides, and protective action of seminal plasma. Fertil. Steril..

[14] Aitken R.J., De Iuliis G.N. (2010). On the possible origins of DNA damage in human spermatozoa. Mol. Human. Reprod..

[15] Hussain T., Kandeel M., Metwally E., Murtaza G., Kalhoro D.H., Yin Y., Tan B., Chughtai M.I., Yaseen A., Afzal A. (2023). Unraveling the harmful effect of oxidative stress on male fertility: A mechanistic insight. Front. Endocrinol..

[16] Mannucci A., Argento F.R., Fini E., Coccia M.E., Taddei N., Becatti M., Fiorillo C. (2022). The Impact of Oxidative Stress in Male Infertility. Front. Mol. Biosci..

[17] Agarwal A., Virk G., Ong C., du Plessis S.S. (2014). Effect of Oxidative Stress on Male Reproduction. World J. Mens. Health.

[18] Aitken R.J. (2017). Reactive oxygen species as mediators of sperm capacitation and pathological damage. Mol. Reprod. Dev..

[19] Ahmadi S., Bashiri R., Ghadiri-Anari A., Nadjarzadeh A. (2016). Antioxidant supplements and semen parameters: An evidence based review. Int. J. Reprod. Biomed..

[20] Stenqvist A., Oleszczuk K., Leijonhufvud I., Giwercman A. (2018). Impact of antioxidant treatment on DNA fragmentation index: A double-blind placebo-controlled randomized trial. Andrology.

[21] Agarwal A., Leisegang K., Majzoub A., Henkel R., Finelli R., Panner Selvam M.K., Tadros N., Parekh N., Ko E.Y., Cho C.-L. (2021). Utility of Antioxidants in the Treatment of Male Infertility: Clinical Guidelines Based on a Systematic Review and Analysis of Evidence. World J. Mens. Health.

[22] Cilio S., Rienzo M., Villano G., Mirto B.F., Giampaglia G., Capone F., Ferretti G., Di Zazzo E., Crocetto F. (2022). Beneficial Effects of Antioxidants in Male Infertility Management: A Narrative Review. Oxygen.

[23] Li K., Yang X., Wu T. (2022). The Effect of Antioxidants on Sperm Quality Parameters and Pregnancy Rates for Idiopathic Male Infertility: A Network Meta-Analysis of Randomized Controlled Trials. Front. Endocrinol..

[24] Dimitriadis F., Borgmann H., Struck J.P., Salem J., Kuru T.H. (2023). Antioxidant Supplementation on Male Fertility—A Systematic Review. Antioxidants.

[25] Osman R., Lee S., Almubarak A., Han J.-I., Yu I.-J., Jeon Y. (2023). Antioxidant Effects of Myo-Inositol Improve the Function and Fertility of Cryopreserved Boar Semen. Antioxidants.

[26] Garolla A., Petre G.C., Francini-Pesenti F., De Toni L., Vitagliano A., Di Nisio A., Grande G., Foresta C. (2022). Systematic Review and Critical Analysis on Dietary Supplements for Male Infertility: From a Blend of Ingredients to a Rationale Strategy. Front. Endocrinol..

[27] Scaruffi P., Licata E., Maccarini E., Massarotti C., Bovis F., Sozzi F., Stigliani S., Dal Lago A., Casciano I., Rago R. (2021). Oral Antioxidant Treatment of Men Significantly Improves the Reproductive Outcome of IVF Cycles. J. Clin. Med..

[28] Cavallini G., Cristina Magli M., Crippa A., Pia Ferraretti A., Gianaroli L. (2012). Reduction in sperm aneuploidy levels in severe oligoasthenoteratospermic patients after medical therapy: A preliminary report. Asian J. Androl..

[29] De Ligny W.R., Fleischer K., Grens H., Braat D.D.M., de Bruin J.P. (2023). The lack of evidence behind over-the-counter antioxidant supplements for male fertility patients: A scoping review. Hum. Reprod. Open..

[30] Adeoye O., Olawumi J., Opeyemi A., Christiania O. (2018). Review on the role of glutathione on oxidative stress and infertility. JBRA Assist. Reprod..

[31] Chen J.Q., Li Y.S., Li Z.J., Lu H.X., Zhu P.Q., Li C.M. (2018). Dietary l-arginine supplementation improves semen quality and libido of boars under high ambient temperature. Animal.

[32] Cheng J.-B., Zhu J., Ni F., Jiang H. (2018). L-carnitine combined with coenzyme Q10 for idiopathic oligoasthenozoospermia: A double-blind randomized controlled trial. Zhonghua Nan Ke Xue.

[33] Xu Z., Liu M., Niu Q.-J., Huang Y.-X., Zhao L., Lei X.G., Sun L.-H. (2023). Both selenium deficiency and excess impair male reproductive system via inducing oxidative stress-activated PI3K/AKT-mediated apoptosis and cell proliferation signaling in testis of mice. Free. Radic. Biol. Med..

[34] Li X., Zeng Y., Luo Y., He J., Luo B., Lu X., Zhu L. (2023). Effects of folic acid and folic acid plus zinc supplements on the sperm characteristics and pregnancy outcomes of infertile men: A systematic review and meta-analysis. Heliyon.

[35] Schisterman E.F., Sjaarda L.A., Clemons T., Carrell D.T., Perkins N.J., Johnstone E., Lamb D., Chaney K., Van Voorhis B.J., Ryan G. (2020). Effect of Folic Acid and Zinc Supplementation in Men on Semen Quality and Live Birth Among Couples Undergoing Infertility Treatment. JAMA.

[36] Conrad M., Moreno S.G., Sinowatz F., Ursini F., Kölle S., Roveri A., Brielmeier M., Wurst W., Maiorino M., Bornkamm G.W. (2005). The Nuclear Form of Phospholipid Hydroperoxide Glutathione Peroxidase Is a Protein Thiol Peroxidase Contributing to Sperm Chromatin Stability. Mol. Cell. Biol..

[37] Kacem O., Harzallah M., Zedini C., Zidi I., Meddeb S., Fékih M., Saidi H., Chaib A., Boughizane S., Ali H.B. (2014). Beneficial Effect of an Oral Antioxidant Supplementation (Fertimax2) on IVF-ICSI Outcomes: A Preliminary Clinical Study. Adv. Reprod. Sci..

[38] Balaban B., Brison D., Calderon G., Catt J., Conaghan J., Cowan L., Ebner T., Gardner D., Hardarson T., Lundin K. (2011). Alpha Scientists in Reproductive Medicine and ESHRE Special Interest Group of Embryology The Istanbul consensus workshop on embryo assessment: Proceedings of an expert meeting. Hum. Reprod..

[39] Gardner D.K., Lane M., Stevens J., Schlenker T., Schoolcraft W.B. (2000). Blastocyst score affects implantation and pregnancy outcome: Towards a single blastocyst transfer. Fertil. Steril..

[40] Mustapha H., Lahimer M., Makni M., Bannour I., Kaabia O., Derouich M., Ferjaoui M.A., Arfaoui R., Zaouali M., Ajina M. (2022). Effect of intrauterine administration of human chorionic gonadotropin one day before fresh blastocyst transfer on clinical outcomes: A quasi-experimental study. Pan Afr. Med. J..

[41] Alahmar A.T. (2022). Coenzyme Q10 improves sperm motility and antioxidant status in infertile men with idiopathic oligoasthenospermia. Clin. Exp. Reprod. Med..

[42] Szymański M., Wandtke T., Wasilow K., Andryszczyk M., Janicki R., Domaracki P. (2021). Comparison of 3- and 6-Month Outcomes of Combined Oral L-Carnitine Fumarate and Acetyl-L-Carnitine Therapy, Included in an Antioxidant Formulation, in Patients with Idiopathic Infertility. Am. J. Mens. Health.

[43] Nateghian Z., Nasr-Esfahani M.H., Talaei-Khozani T., Tavalaee M., Aliabadi E. (2023). L-Carnitine and Pentoxifylline Supplementation Improves Sperm Viability and Motility at Low Temperature. Int. J. Fertil. Steril..

[44] Micic S., Lalic N., Djordjevic D., Bojanic N., Bogavac-Stanojevic N., Busetto G.M., Virmani A., Agarwal A. (2019). Double-blind, randomised, placebo-controlled trial on the effect of L-carnitine and L-acetylcarnitine on sperm parameters in men with idiopathic oligoasthenozoospermia. Andrologia.

[45] Jannatifar R., Parivar K., Roodbari N.H., Nasr-Esfahani M.H. (2019). Effects of N-acetyl-cysteine supplementation on sperm quality, chromatin integrity and level of oxidative stress in infertile men. Reprod. Biol. Endocrinol..

[46] Jannatifar R., Asa E., Sahraei S.S., Verdi A., Piroozmanesh H. (2022). N-acetyl-l-cysteine and alpha lipoic acid are protective supplement on human sperm parameters in cryopreservation of asthenoteratozoospermia patients. Andrologia.

[47] Tunc O., Thompson J., Tremellen K. (2009). Improvement in sperm DNA quality using an oral antioxidant therapy. Reprod. BioMedicine Online.

[48] Tremellen K., Miari G., Froiland D., Thompson J. (2007). A randomised control trial examining the effect of an antioxidant (Menevit) on pregnancy outcome during IVF-ICSI treatment. Aust. N. Zeal. J. Obstet. Gynaecol..

[49] Yaris M., Akdogan N., Öztürk M., Bozkurt A., Karabakan M. (2022). The effects of two different antioxidant combinations on sperm parameters. Urologia.

[50] Lafuente R., González-Comadrán M., Solà I., López G., Brassesco M., Carreras R., Checa M.A. (2013). Coenzyme Q10 and male infertility: A meta-analysis. J. Assist. Reprod. Genet..

[51] Cassuto N.G., Piquemal D., Boitrelle F., Larue L., Lédée N., Hatem G., Ruoso L., Bouret D., Siffroi J.-P., Rouen A. (2021). Molecular Profiling of Spermatozoa Reveals Correlations between Morphology and Gene Expression: A Novel Biomarker Panel for Male Infertility. BioMed Res. Int..

[52] Moretti E., Signorini C., Noto D., Corsaro R., Collodel G. (2022). The relevance of sperm morphology in male infertility. Front. Reprod. Health.

[53] Ribas-Maynou J., Novo S., Torres M., Salas-Huetos A., Rovira S., Antich M., Yeste M. (2022). Sperm DNA integrity does play a crucial role for embryo development after ICSI, notably when good-quality oocytes from young donors are used. Biol. Res..

[54] Cooper T.G., Noonan E., von Eckardstein S., Auger J., Baker H.W.G., Behre H.M., Haugen T.B., Kruger T., Wang C., Mbizvo M.T. (2010). World Health Organization reference values for human semen characteristics. Human. Reprod. Update.

[55] Agarwal A., Gupta S., Sharma R.K. (2005). Role of oxidative stress in female reproduction. Reprod. Biol. Endocrinol..

[56] Agarwal A., Baskaran S., Parekh N., Cho C.-L., Henkel R., Vij S., Arafa M., Panner Selvam M.K., Shah R. (2021). Male infertility. Lancet.

[57] Menezo Y., Clement P., Amar E. (2017). Evaluation of sperm DNA structure, fragmentation and decondensation: An essential tool in the assessment of male infertility. Transl. Androl. Urol..

[58] Delbarba A., Arrighi N., Facondo P., Cappelli C., Ferlin A. (2020). Positive effect of nutraceuticals on sperm DNA damage in selected infertile patients with idiopathic high sperm DNA fragmentation. Minerva Endocrinol..

[59] Marinaro J.A., Schlegel P.N. (2023). Sperm DNA Damage and Its Relevance in Fertility Treatment: A Review of Recent Literature and Current Practice Guidelines. Int. J. Mol. Sci..

[60] Robinson L., Gallos I.D., Conner S.J., Rajkhowa M., Miller D., Lewis S., Kirkman-Brown J., Coomarasamy A. (2012). The effect of sperm DNA fragmentation on miscarriage rates: A systematic review and meta-analysis. Human. Reprod..

[61] Smith G.D., Takayama S., Swain J.E. (2012). Rethinking In Vitro Embryo Culture: New Developments in Culture Platforms and Potential to Improve Assisted Reproductive Technologies. Biol. Reprod..

[62] Zarbakhsh S. (2021). Effect of antioxidants on preimplantation embryo development in vitro: A review. Zygote.

[63] Łakoma K., Kukharuk O., Śliż D. (2023). The Influence of Metabolic Factors and Diet on Fertility. Nutrients.

[64] Wyck S., Herrera C., Requena C.E., Bittner L., Hajkova P., Bollwein H., Santoro R. (2018). Oxidative stress in sperm affects the epigenetic reprogramming in early embryonic development. Epigenetics Chromatin.

