# Supplementary material for: Effect of Micronutrients and L-Carnitine as Antioxidant on Sperm Parameters, Genome Integrity, and ICSI Outcomes: Randomized, Double-Blind, and Placebo-Controlled Clinical Trial
Source: Antioxidants (Basel). 2023 Oct 31;12(11):1937. doi: 10.3390/antiox12111937 (PMC10669279; doi:10.3390/antiox12111937)
Supplement: Supplementary file 1 [file antioxidants-12-01937-s001.zip › antioxidants-2579497-supplementary.pdf]

Table S1. The table below illustrates the variations in the follow-up period, depending on the couples' status. For couples who achieved a live birth, the follow-up was terminated at the time of delivery, resulting in an average follow-up duration of 433 days (with a median of 444.5 days). In contrast, couples who did not achieve a live birth had an average follow-up period of 557 days, approximately 18 months, but with a median of 710.5 days, approximately 23.6 months. These results show no difference between the two groups.

Treatment	Pregnancy	Mean	n	sd	min	max	median
Fertilis	No	553.71	114	189.5	237	730	710.5
	yes	445.0	17	80.4	346	589	468.0
	Total	539.6	131	182.7	237	730	491.0
Placebo	No	559.6	125	192.07	233	730	730.0
	Yes	403.0	7	93.1	304	549	356.0
	Total	551.3	132	191.2	233	730	710.5
Total	No	556.8	239	190.5	233	730	730.0
	Yes	432.7	24	84.5	304	589	444.5
	total	545.5	263	186.7	233	730	569.0
